# Annotations

**Published:** 1905-09-09

**Authors:** 


### ANNOTATIONS.

### The Shadow of Cholera.

There was a time when the mention of the word cholera excited the apprehensions of the nervous. To-day the tendency is rather in the other direction. We have enjoyed immunity from this formidable disease for such a long period that people are apt to suppose that England is proof against it. But it is just as well that those who seem to be under the impression that cholera is not in the least likely to invade these shores again, should understand that there are periods when it can only be kept out by the most vigilant measures. We do not say that such a season has arrived, but the undoubted prevalence of cholera in several parts of Europe is, at all events, a sufficient reason why scrupulous care should be exercised by the authorities at our own ports to guard against the introduction here of a scourge which it is absurd to think would not speedily spread if once it gained admission. There are still the conditions for a serious epidemic in most of our large cities, and safety lies only in absolute prevention. It is satisfactory to learn that so far the situation in Hamburg, not many years ago the centre of an extensive outbreak, is not very alarming, but so long as in other parts of Germany the disease continues to attack fresh persons, the utmost watchfulness will be required by all whose duty it is to save this country from its ravages. We are glad that the Local Government Board have promptly issued a circular to the port authorities which proves that they are fully alive to the importance of energetic precautionary measures, especially with respect to the movements of foreign emigrants.

### Guardians and the Sick Poor.

People who have to do with the administration of poor-law relief in London, where as a rule every consideration is shown for genuine distress, can hardly realise how differently—more, we think, from thoughtlessness than from hardness of heart—cases are treated in some parts of the country. Thus, the Wolverhampton Guardians have resolved: "That in future all applicants for medical attendance (except persons in receipt of out-relief) be required to attend the meeting of the Board (for relief applications) next after the application is made." This resolution, on the face of it, cannot be carried out literally. The applicant may be too ill to appear at the meeting of the board next after his application. If the application be for some member of his family, the thing is not physically impossible, but it may be morally very unreasonable. If a man has obtained work since making an application for medical relief for some member of his family, it would be unkind to ask him to neglect it, and perhaps lose it in consequence, in order to appear before the guardians. It might be possible for some other representative of the family to appear, but in households where free medical treatment is needed, the wife or mother, who is the only other responsible person, can rarely be spared from attendance on the invalid's sick-bed. It seems to us unreasonable to ask for this attendance during the time of sickness. Probably the reason why the guardians wish to see the applicant is to make such inquiries as would make it clear to them whether or not the relief asked for should be given on loan. This is a very reasonable thing to do, and even when the inquiry results in the relief being given free, it is well that the applicant should be made to realise that the assistance given is not to be had too lightly. But in most cases the relieving officer can elicit sufficient information about the family circumstances to guide the guardians in deciding whether or not to make a charge. If the decision is that the relief be given on loan, the applicant, or someone representing him, can appear at a subsequent meeting to make any explanation or appeal regarding it. In many cases it would be possible, after the illness is past, for the family to make a contribution, which it would be cruel to insist on while the expenses of sickness had to be met, so that in the end the parish would not lose by delay. The principle of making the applicant appear before the Guardians is in itself a good one, but unless a certain amount of flexibility is allowed as to the time of appearing—a matter which might be left to the discretion of the relieving officer—the result will be in every way unsatisfactory.

### Open-Air Treatment in Towns.

The advisability of securing abundance of pure fresh air as a valuable therapeutic factor in the treatment of disease, though not appreciated by the public, is now widely recognised by the medical profession. As a result, ingenious minds are ever at work regarding the means by which the desired end can be best attained. In more than one quarter it has been proposed to move a considerable section of the hospital population into the country, or, at all events, to establish country hospitals for certain forms of disease. How far this may be possible in the future it is difficult to say, but it is by no means impossible that increased rapidity and ease of transit may solve one of the difficulties of the scheme—namely, the provision of efficient medical attendance. In the meantime, however, disease exists, and it is the duty of the profession to secure for its victims the influences best calculated to obtain restored health. When the best is for various reasons impossible the wise man is not content merely to sigh for the unattainable; on the contrary, he makes the most of what is at hand. It is this spirit which has encouraged the staffs of various hospitals in different parts of the country to cultivate the development in connection with hospital wards of balconies where patients may spend the greater part, or even the whole, of the day. Even in large towns where the air is not of the purest, this has been found an advantage, and it applies not merely to convalescents but also to cases of severe and acute illness, as for example, pernicious and other grave forms of anæmia. The principle might be more widely adopted, and we should like to see it generally recognised. In London, noise and dirt are serious factors, but with a little ingenuity these may be minimised and, in any case, the value of fresh air remains.

